# Revealing Microbiome Structure and Assembly Process in Three Rhizocompartments of *Achyranthes bidentata* Under Continuous Monoculture Regimes

**DOI:** 10.3389/fmicb.2021.677654

**Published:** 2021-06-14

**Authors:** Juanying Wang, Hongmiao Wu, Linkun Wu, Ye Liu, Puleng Letuma, Xianjin Qin, Ting Chen, Christopher Rensing, Sheng Lin, Wenxiong Lin

**Affiliations:** ^1^Fujian Provincial Key Laboratory of Agroecological Processing and Safety Monitoring, College of Life Sciences, Fujian Agriculture and Forestry University, Fuzhou, China; ^2^Key Laboratory for Genetics, Breeding and Multiple Utilization of Crops, Ministry of Education, College of Crop Sciences, Fujian Agriculture and Forestry University, Fuzhou, China; ^3^Department of Crop Science, National University of Lesotho, Maseru, Lesotho; ^4^College of Crop Sciences, Fujian Agriculture and Forestry University, Fuzhou, China; ^5^Fujian Provincial Key Laboratory of Soil Environmental Health and Regulation, College of Resources and Environment, Fujian Agriculture and Forestry University, Fuzhou, China

**Keywords:** co-occurrence network, rhizosphere, rhizoplane, root, plant–soil interactions

## Abstract

The complex composition and interaction of root-associated microbes are critical to plant health and performance. In this study, we presented a detailed characterization of three rhizocompartment (rhizosphere, rhizoplane, and root) microbiomes of *Achyranthes bidentata* under different years of consecutive monoculture by deep sequencing in order to determine keystone microorganisms *via* co-occurrence network analysis. The network analysis showed that multiple consecutive monoculture (MCM, represented 5Y and 10Y) soils generated some distinct beneficial bacterial taxa such as *Bacillus*, *Fictibacillus*, *Bradyrhizobium*, *Shinella*, and *Herbaspirillum*. For fungi, *Mortierella* substituted for *Fusarium* in occupying an important position in different rhizocompartments under *A. bidentate* monoculture. Quantitative PCR analysis confirmed a significant increase in *Bacillus*, *Pseudomonas*, and *Burkholderia* spp. The results of the inoculation assay showed that addition of beneficial bacteria *Bacillus subtilis* 74 and *Bacillus halodurans* 75 significantly increased the root length and fresh weight of *A. bidentata.* Furthermore, three types of phytosterones, as the main allochemicals, were identified both in the rhizosphere soil and in culture medium under sterile conditions by LC-MS/MS. When looking at *in vitro* interactions, it was found that phytosterones displayed a positive interaction with dominant beneficial species (*Bacillus amyloliquefaciens* 4 and *B. halodurans* 75) and had a negative effect on the presence of the pathogenic fungi *Fusarium solani* and *Fusarium oxysporum*. Overall, this study demonstrated that consecutive monoculture of *A. bidentata* can alter the bacterial and fungal community by secreting root exudates, leading to recruitment of beneficial microbes and replacement of plant-specific pathogenic fungi with plant beneficial fungi.

## Introduction

Chinese medicine resources are a key point for human disease treatment. *Rehmannia glutinosa*, *Radix pseudostellariae*, and *Achyranthes bidentata* are perennial herbaceous plants that are highly valued in traditional Chinese medicine with a surging market demand (Li et al., 2015; Sun et al., 2015; Wu et al., 2017). However, many medicinal plant species, such as *R. glutinosa* and *R. pseudostellariae*, are grown under a continuous cropping system in a geo-authentic production zone because the synthesis of bioactive constituents of certain Chinese medicinal plants is closely correlated to ecological factors (Zhang et al., 2010). In general, this system can have a detrimental effect on soil quality leading to a significant decline in plant biomass and vigor as well as increasing occurrence of plant pests (Wu et al., 2015, 2019). Such “replanting disease” have seriously affected the sustainable development of medicinal plant production (Qian et al., 2016). In contrast, *A. bidentata* (family *Amaranthaceae*) is very suitable for consecutive monoculture (Hao et al., 2008; Chen et al., 2015; Wang et al., 2019). Interestingly, the plant biomass and the main active ingredients (includes phytosterone and saponin) in monoculture plots were higher than those in newly planted plots (Li et al., 2010). There is an urgent need to fully understand the complex reasons why different crops respond differently to continuous cropping in the managed farming system.

Numerous studies have suggested that below-ground microbes play crucial roles in soil quality and plant health (Macdonald and Singh, 2013; Lakshmanan et al., 2014). Wu et al. (2015) found that *R. glutinosa* monoculture led to a serious decline in tuberous root biomass, which was associated with a significant increase in the amount of *Fusarium oxysporum*, and a decrease in beneficial *Pseudomonas* spp. in the rhizosphere. In addition, previous studies demonstrated that the occurrence of soil-borne diseases was responsible for either a decrease in soil microbial diversity or an increase in population size of antagonistic bacteria (Latz et al., 2012; Shen et al., 2014). However, our research (Wang et al., 2019) found that in rhizosphere soil, beneficial groups conferring “biocontrol” effects on plant growth increased with increasing years of *A. bidentata* planting. Therefore, the structure of below-ground microbial community was shown to be a determining constraint to consecutive monoculture.

The environment including the soil and plant root can be divided into three compartments: endosphere (ER, inside the root), rhizoplane (RP, the soil on the surface of the root), and rhizosphere (RS, the soil around the root) (Edwards et al., 2015), which are considered as ecological niches colonized by different microorganisms that play different roles in the soil ecosystem. It was noted that the established endosphere microbiome contributes significantly to several functional characteristics in plants including immune system modulation, quorum sensing, and type V and VI protein secretion systems (Barbara et al., 2015). Moreover, the rhizoplane was shown to play a critical gating role for controlling microbial entry into the host tissue (Ofek-Lalzar et al., 2013). It has been documented that more intimate microbe–host interactions occurred and a more specialized community was further enriched on the rhizoplane than the rhizosphere (Zhang et al., 2017). Studies have indicated that the microbial community in the rhizosphere contained up to 10^11^ microbial cells per gram of root (Egamberdieva et al., 2007) and more than 30,000 prokaryotic species (Mendes et al., 2011), and these were referred to as “the second genome” of the plant (Berendsen et al., 2012; Rout and Southworth, 2013). The narrow zone rhizosphere was also referred to as a fierce battleground for microorganisms competing for plant-derived nutrients (Martínez-Viveros et al., 2010). Previous studies have revealed that plant species and genotypes alongside environmental and edaphic factors determine the distribution and diversity of the three root-associated microbiota (Van der Heijden and Schlaeppi, 2015; Poudel et al., 2019). Among them, root exudates are vital for the interactions between roots and microbes. It has been established that plants recruit plant-beneficial microbes by secretion of the exudates from their roots in response to an attack. For example, root exudates of *Arabidopsis* were shown to enhance colonization by a growth-promoting bacterium (*Bacillus cereus*) with subsequent chitinase production; consequently, the PGPR *B. cereus* was able to significantly increase biomass and induce systemic resistance in *A. thaliana* (Zhou et al., 2015). However, there is limited information on the microbial community changes of three root-associated compartments (endosphere, rhizoplane and rhizosphere) in *A. bidentata* under monoculture and the ecological effects of root exudates.

Recently, with the development of high-throughput sequencing and bioinformatic analysis, studies on the co-occurrence network analysis have provided new insights into the bacterial and fungal composition, distribution, and microbial interaction patterns in environments ranging from soil (Berendsen et al., 2012; Ma et al., 2016) to water (Hu et al., 2017; Mikhailov et al., 2019) and even to human gut (Li et al., 2019). Unlike traditional analytical approaches, network analysis-based approaches, as measured by simultaneous changes in pairwise taxa abundance, can disentangle the structure and assembly of the microbial community, map the pathways of potential interactions between microorganisms, and offer meaningful information beyond sample-level comparison (Cardinale et al., 2015, 2020; Jiang et al., 2017). Based on this analysis, researchers were also able to identify the keystone microbial groups that have the largest influence on community structure and possible function regardless of their population size (Lupatini et al., 2014; Araya et al., 2020). Moreover, the analysis can yield a holistic view of linkages between microbial groups and environmental variables (Banerjee et al., 2018; Fan et al., 2018).

This study aimed to analyze the fungal and bacterial communities associated with the rhizosphere, rhizoplane, and endosphere of *A. bidentata* using high-throughput sequencing. We hypothesized that the microbial community composition and network structure would differ from each other in the three rhizocompartments, thereby exhibiting their different eco-niche functions in soil ecosystem due to distinct microenvironment filtering, and response to consecutive monoculture. Furthermore, we aimed to isolate key microorganisms so that they could be applied to verify the relationship with root exudates. Therefore, this study will not only enhance the understanding in ecological interactions of plant associated bacterial and fungal communities, but is intended to lay the foundation for further exploitation and application of this core community for sustainable agriculture.

## Materials and Methods

### Field Sites

The experiment was performed at a long-term orientation station of Fujian Agriculture and Forest University (FAFU) located in Xitao town (34.99 N, 113.26 E), Wuzhi, Henan Province, China. The test plant material was *A. bidentata* cultivar “Hetaowen,” which is common in the main production region. *A. bidentata* was planted in the last 10 days of June and harvested in the middle of November in the same year. After harvest, fields were cultivated with wheat from December to June of the following year. The six soil treatments were as follows: (i) newly planted (1Y), (ii) 2-year consecutive monoculture (2Y), (iii) 3-year consecutive monoculture (3Y), (iv) 5-year consecutive monoculture (5Y), (v) 10-year consecutive monoculture (10Y), and (vi) control with no *A. bidentata* plant (CK).

### Sample Preparation

The tuberous roots (at about 40 cm deep) were harvested at the most productive phase of plant development (on November 5, 2017) to evaluate the microbial communities. Samples from each treatment were collected by a five-point sampling method. Briefly, five plots (2 m ^∗^ 2 m) were selected randomly in each treatment. From each plot, three plants were dug out from the soil and then proceeded by shaking off the bulk soil from the root. The three root-associated compartment samples were collected by the method described by Edwards et al. (2015). Briefly, approximately 1 mm of rhizosphere (RS) soil attached to the roots was collected. The rhizoplane (RP) soil was then obtained after sonicating the root for 30 s at 50–60 Hz in a sterile container with sufficient sterile phosphate buffered saline (PBS) solution. Then, the roots were used to extract the root (RT) microbiome. Root microbiome represented the microorganisms attached to and inside the root, which included the endosphere microbiome. The samples obtained from each treatment were mixed and then divided into three parts for analysis.

Each rhizocompartment sample was then stored at −80°C until DNA extraction. The physicochemical characteristics of air-dried RS soil was assayed in three technical replicates as described by Wu et al. (2013). Briefly, the total N, P, and K (TN, TP, and TK) was calculated by first digesting the soil using the H_2_SO_4_–H_2_O_2_. Then, TN was measured using the Kjeldahl method. TP and Available P (AP, extracted with NaHCO_3_ solution) were examined by a colorimetric method. TK and Available K (AK, extracted in ammonium acetate) were determined by flame atomic absorption spectrometry. Available N (AN) was calculated by converting available nitrogen into ammonia. Soil pH was measured by using a pH meter in a 1:2.5 soil/water suspension.

### DNA Extraction, PCR Purification, and High-Throughput Sequencing

Total DNA were extracted from 0.7 g of soil samples and 0.5 g of roots using BioFast Soil Genomic DNA Extraction kit (BioFlux, Hangzhou, China) according to the manufacturer’s instructions. DNA concentration was detected by using Nanodrop 2000C Spectrophotometer (Thermo Fisher Scientific, United States). For the bacterial community, the V4 amplification of the 16S rRNA gene in RS and RP samples was carried out with the specific primers 515F (5′-GTGCCAGCMGCCGCGGTAA-3′) and 806R (5′-GGACTACHVGGGTWTCTAAT-3′) (Panke-Buisse et al., 2014). Variable regions 5 to 7 (V5–V7) in RT samples were selected and amplified with the specific primers 799F (5′-AACMGGATTAGATACCCKG-3′) and 1193R (5′-ACGTCATCCCCACCTTCC-3′) with the barcode (Beckers et al., 2016). For the fungal community, the primer set ITS5-1737F (5′-GGAAGTAAAAGTCGTAACAAGG-3′)/ITS2-2053R (5′-GCTGCGTTCTTCATCGATGC-3′) for RS and RP samples (Lu et al., 2013) and the primer set ITS1-1F-F (5′-CTTGGTCATTTAGAGGAAGTAA-3′)/ITS2-2053R (5′-GCTGCGTTCTTCATCGATGC-3′) for RT samples (Xiong et al., 2016) were chosen to target the fungal ITS1 region. The primer pairs selected in RT samples can effectively filter mitochondrial DNA or plastid (chloroplast) DNA, thereby improving the accuracy of the results. The PCR reactions were carried out by Novogene Co., Ltd. (Beijing, China) using Phusion^®^ High-Fidelity PCR Master Mix with GC Buffer (New England Biolabs, Ipswich, United States). The PCR products were purified with Qiagen Gel Extraction Kit (Qiagen, Germany). The library was then sequenced on an Illumina HiSeq2500 platform. The sequences were submitted to the NCBI database under the accession number PRJNA606926.

### Sequence Data and Co-occurrence Network Analyses

Raw sequences were processed using the Quantitative Insights Into Microbial Ecology (QIIME) toolkit (V1.9.1^1^) (Caporaso et al., 2010) and UPARSE pipeline (Uparse v7.0.1001^2^) (Edgar, 2013). Then Usearch software was used to perform the operations of data filtering chimeras and clustering to generate operational taxonomic units (OTUs) at the 97% similarity level. Species annotation analysis was performed (set threshold value 0.8–1) using Mothur method and the SSUrRNA database of SILVA (v132^3^) to obtain taxonomic information and each classification level (Wang et al., 2007; Quast et al., 2012). The co-occurrence network analysis was implemented to demonstrate the relationship of different species among several samples and find out the keystone species (Li and Wu, 2018). The co-occurrence patterns in this study were performed using the 50 most abundant genera of bacterial and fungal communities based on strong (*r* > 0.6) and significant Spearman correlations (*p* < 0.01) in R software. The R scripts are found at https://github.com/RichieJu520/Co-occurrence_Network_Analysis. Moreover, the networks visualization, topological properties, and modular analysis were performed in Gephi. Heatmap was used to perform the correlations between soil physical–chemical properties and the keystone species in the network.

### Quantitative PCR *in situ* for Dominant Genera in the Consecutive Monoculture Rhizosphere Soil

A quantitative PCR *in situ* was used to determine the variation tendency of total bacteria (Eub338/Eub518), total fungi (ITS1F/ITS4), and dominant genera and species including *Pseudomonas* spp. (Ps-for/Ps-rev), *Burkholderia* spp. (Burk3/BurkR), *Bacillus* spp. (BacF/1378), and *F. oxysporum* (ITS1F/AFP308R) using the CFX96 Real-Time system (Bio-RDA, United States) in rhizosphere soil (Lievens et al., 2005; Drigo et al., 2009). The mixture volume of PCR reaction was 15 μl containing 7.5 μl of 2 × TransStart Tip Green qPCR Supermix (TransGen, Beijing, China), 0.6 μl of each primer (10 μmol L^–1^), 5.3 μl of RNase-free H_2_O, and 1 μl of template DNA (20 ng μl^–1^ of total soil DNA or a serial dilution of plasmid DNA for standard curves). Four independent quantitative PCR assays were performed for each treatment.

### Identification of the Main Substance Phytosterones in Culture Medium and Rhizosphere Soil and Its Interaction With Specific Bacterium *in vitro*

#### LC-MS/MS Analysis of Phytosterones in Different Rhizosphere Soil

Tissue cultures of *A. bidentata* were incubated for 44, 140, 248, 294, 320, 373, and 396 days. After removing the plants root, 90 ml of culture medium was collected and extracted with 50% aqueous methanol and dissolved in 2 ml of methanol. Soil phytosterones of each sample were extracted with 70% aqueous methanol and dissolved in 4 ml of methanol. The extraction procedure of phytosterones and detection using LC-MS/MS was previously described by Wang et al. (2019).

#### Screening and Functional Verification of the Specific Beneficial Bacteria

*Bacillus* spp. in soil were isolated on nutrient agar (NA) media following the methods of Ahmad et al. (2008). A total of 200 isolates were selected for further *in vitro* antagonism assays. Monitoring the antagonistic activity of single colonies of bacteria against *F. oxysporum* and *F. solani* were recorded by the method described by Wu et al. (2015). Furthermore, the 16S rRNA gene of *Bacillus* isolates were amplified by universal primers 27F/1522R and the PCR products were subsequently visualized on an agarose gel and sequenced by Shanghai Biosune Co. Ltd (Shanghai, China). The results were compared to the sequences on NCBI using the BlastN. The sequences were submitted to the NCBI database under accession number SUB9495955.

The pots (13 cm bottom diameter, 18 cm top diameter, and 24 cm height) containing soil were sown with the seeds of *A. bidentata* and placed in a greenhouse on March 5, 2018. The isolated bacteria *Bacillus amyloliquefaciens* 4, *Bacillus subtilis* 35, *B. subtilis* 74, and *Bacillus halodurans* 75 were grown in LB medium at 37°C and 200 rpm, respectively. When the OD_600_ value reached 0.8, 10 ml of bacterial fermented broth was centrifuged and washed with double-distilled water twice after removing the supernatant. The cells with equal volume of double-distilled water were added into the soil at the four-leaf stage of *A. bidentata*; meanwhile, the same volume of double-distilled water was added to the control. The treatment was repeated four times on April 7, May 7, May 20, and June 7, 2018, respectively. Each treatment had three replicates. We harvested the plants on July 15, and immediately measured plant height, root length, and fresh weight.

#### *In vitro* Interaction Between the Beneficial Bacterium and Specific Phytosterones

Based on the measurement results of phytosterones in the *A. bidentata* rhizosphere soil, a concentration gradient series of single phytosterone and mixed phytosterone (β*-ecdysterone*:25R-*inokosterone*:25S-*inokosterone* = 20:2:3) were prepared at 0, 0.01, 0.05, 0.1, 0.5, 1, and 2 μg/ml. The isolated bacterial BA 4, BS 35, BS 74, and BH 75 strains were cultured by adding the single or mixed phytosterone to LB medium with eightfold dilution. After 8 h of incubation at 30°C and shaking at 200 rpm, the optical density (OD) values were determined at 600 nm using a microplate reader (Thermo Scientific Multiskan MK3, Shanghai, China). The isolated *F. oxysporum* or *F. solani* was inoculated onto the center of 9-cm-diameter Petri dishes filled with an eightfold dilution of potato–sugar–agar (PSA) medium containing different final concentrations of single or mixtures of phytosterone (0, 0.01, 0.05, 0.1, 0.5, 1, and 2 μg/ml). After 7 days of incubation at 30°C, colony diameter was measured.

### Statistical Analyses

Alpha diversity analyses including Observed-OTUs, Shannon index, Simpson index, Chao1, and ACE were conducted according to the normalized data by QIIME (V1.9.1) (Caporaso et al., 2010). Venn diagrams of the exclusive and shared OTUs between different samples were displayed with R software (version 3.0.3). Analysis of variance (ANOVA) was conducted for significance of difference by Tukey’s test (*p* < 0.05) using DPS 7.05 software.

## Results

### The Morphology and Biomass of *A. bidentata* Under Consecutive Monoculture

The observation of morphology and measurement of yield showed plants displayed well with less adventitious fibrous roots and more below-ground biomass when *A. bidentata* were harvested from consecutive monocultured soil in the period of tuber enlargement. Additionally, compared with results from a newly planted plot (1Y), the dry weight of *A. bidentata* tuberous roots displayed a 2.19 times increase in 10 years monoculture plot (10Y) (Supplementary Figure 1).

### Assessment of Physicochemical Properties in the Rhizosphere Soil

Physicochemical properties of rhizosphere soil samples were summarized in Supplementary Table 1. The results revealed that compared to the less continuously planted plot (1Y and 3Y), the contents of total phosphorus (TP) and available nitrogen (AN) in rhizospheric soils were significantly (*p* < 0.05) higher in the multiple continuous monoculture soil systems (5Y and 10Y). Meanwhile, the pH value does not decrease with increasing years of monoculture. However, the newly planted soil had a higher total potassium (TK) than the monoculture soils. It was found that the total nitrogen (TN) displayed a slightly increasing trend in the monoculture system even if there was no significant difference.

### Diversity Indices of the Soil Microbial Community

The results generated 885,359, 888,537, and 611,754 high-quality effective tags of each root-associated layer with a length of 253 bp (RS, RP) and 376 bp (RT), and tags were clustered to 45,959, 52,378, and 7192 bacterial OTUs, respectively. With respect to fungi, a total of 650,006, 865,696, and 850,159 high-quality ITS1 effective tags were obtained with a length of 260 bp (RS, RP) and 250 bp (RT). Subsequently, at a 97% sequence similarity cutoff, the tags were clustered to 8938, 6166, and 3553 fungal OTUs across the 12 samples. The alpha-diversity indices of bacterial and fungal communities were observed including Shannon index, Simpson index, Chao1, and ACE (Supplementary Tables 2, 3). It can be seen that the Chao1 and ACE index of bacteria and fungi displayed a trend in the order rhizosphere > rhizoplane > root. Further, it was found that the Simpson, Chao1, and ACE indices of bacterial and fungal communities were not significantly different to different continuous cropping years in the rhizosphere and rhizoplane. However, the number of OTUs and ACE indices of the bacterial community increased with increasing years of monoculture in root. The reverse was true with respect to fungal community (Supplementary Table 3).

### Venn Diagram Analysis

The exclusive and shared OTUs between different samples were presented by Venn diagrams (Supplementary Figures 2, 3). Interestingly, for bacterial communities, the number of OTUs shared in 5Y and 10Y samples was 519, 214, and 73 from rhizosphere to root and higher than those shared in 1Y and 3Y samples (285, 190, and 22, respectively). It can be seen from the pie chart structure that the microorganisms shared by 5Y and 10Y are completely different from those between 1Y and 3Y samples. Moreover, the OUTs shared in 5Y and 10Y in the rhizosphere layer (Supplementary Figure 2a) were mainly assigned to the class *Proteobacteria* (28.5%), *Planctomycetes* (16.6%), and *Bacteroidetes* (13.1%), while in root (Supplementary Figure 2c), the *Proteobacteria* (58.9%) accounted for more than half of the total OTUs. For fungal communities (Supplementary Figure 3), the number of shared OTUs in 5Y and 10Y samples was less than those in 1Y and 3Y samples, which was opposite the trend displayed in bacteria except RS. Accordingly, the number of OTUs shared in 5Y and 10Y samples was 17 and 3 in rhizoplane and root, which are by far lower than those shared in 1Y and 3Y samples (101 and 57). The OTUs shared in 1Y and 3Y classified at the phylum level were similar in these three root-associated layers.

### Structure and Composition of Soil Microbial Community

**FIGURE 1:**
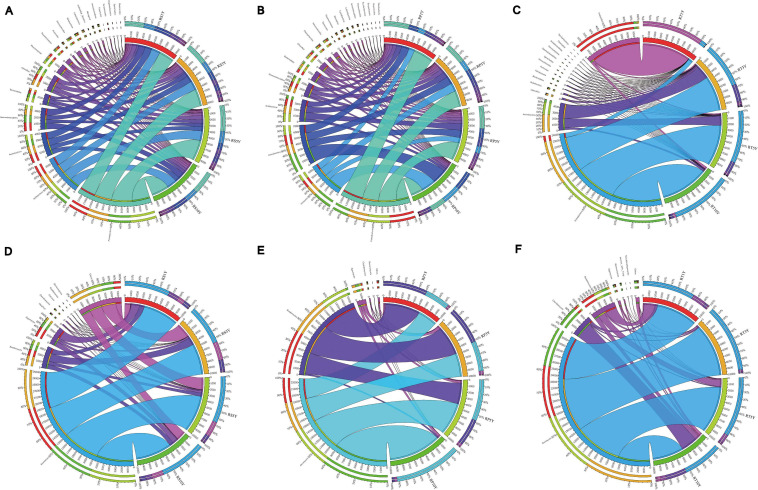
Distribution of dominant (top 20) phyla in four different samples in three root-associated compartments. [**(A–C)** represented the bacterial community in rhizosphere, rhizoplane, and root, respectively; **(D–F)** represented the fungal community in rhizosphere, rhizoplane, and endosphere, respectively]. The thickness of each ribbon represents the abundance of each taxon. The relative tick above the outer segment stands for the relative abundance of each taxon. The data were visualized using Circos (Version 0.69, http://circos.ca/).

**FIGURE 2:**
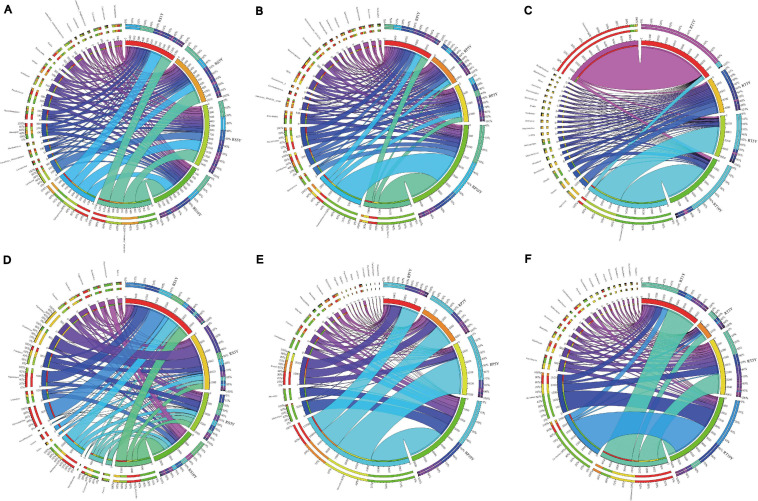
Distribution of dominant (top 20) genera in four different samples in three root-associated compartments. [**(A–C)** represent the bacterial community in rhizosphere, rhizoplane, and root, respectively; **(D–F)** represented the fungal community in rhizosphere, rhizoplane, and root, respectively]. The thickness of each ribbon represents the abundance of each taxon. The relative tick above the outer segment stands for the relative abundance of each taxon. The data were visualized using Circos (Version 0.69, http://circos.ca/).

As shown in Figures 1, 2, the proportions of various phyla and genus were different across the compartments. The bacterial OTUs in RP were predominantly associated with five phyla, *Proteobacteria*, *Firmicutes*, *Actinobacteria*, *Acidobacteria*, and *Gemmatimonadetes*, and accounted for 80.8%, 80.9%, 77.7%, and 86.8% of the total population in the 1Y, 3Y, 5Y, and 10Y sample, respectively. *Proteobacteria* was the dominant microbial taxon in RT samples (Figures 1B,C). It is worth mentioning that each of the compartments was found to harbor a distinct microbiome, and the most relative abundance *Bacillus* (RP) by the phyla *Firmicutes* and *Pseudomonas* (RT) belong to *Proteobacteria* (more than 50% in most samples) increased with the increasing years of monoculture (Figures 2A,B). Fungal OTUs were comprised mainly of the phyla *Ascomycota*, *Basidiomycota*, and *Zygomycota*, and these three phyla accounted for exceeding 70% in each sample of each layer. Other minor phyla including *Chytridiomycota*, *Rozellomycota*, and *Glomeromycota* were mostly found in the RS compartments (Figures 1D–F). Variation in community composition among the three plant compartments was also observed at the genus level (Figures 2D–F). The three most abundant genera (*Mortierella*, *Microidium*, and *Cladosporium*) showed different patterns of relative abundance. Further, the trends of *Mortierella* (*Mucoromycota*) were similar in each plant compartment, and the proportion in 10Y was higher than those in the other soil samples (1Y, 3Y, and 10Y). Although the content of *Fusarium* (*Ascomycota*) was increased with increasing years of monoculture in RS and RP, the proportion was lower in 10Y compared to 1Y in RT.

### Co-occurrence Network Analysis

**FIGURE 3:**
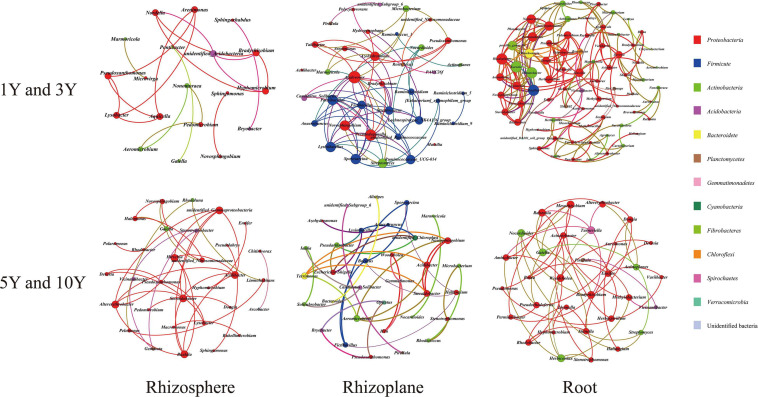
The co-occurrence network of bacterial genera based on correlation analysis for different samples in three root-associated compartments. A connection stands for a strong (Spearman’s *r* > 0.6) and significant (*p* < 0.01) correlation. The size of each node is proportional to the degree. Nodes colored by taxonomy.

**FIGURE 4:**
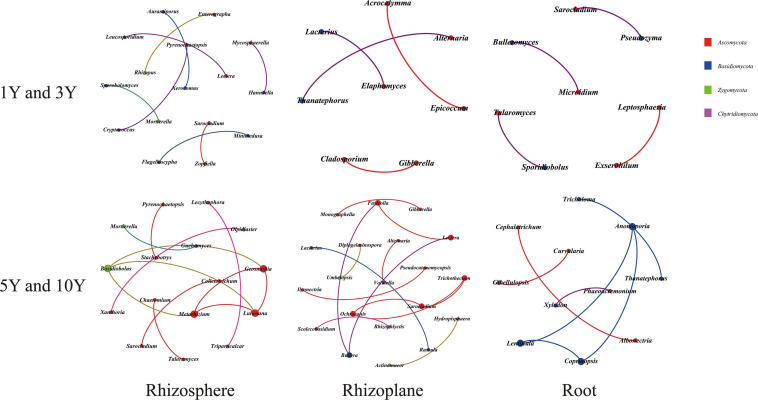
The co-occurrence network of fungal genera based on correlation analysis for different samples in three root-associated compartments. A connection stands for a strong (Spearman’s *r* > 0.6) and significant (*p* < 0.01) correlation. The size of each node is proportional to the degree. Nodes colored by taxonomy.

In this study, 12 networks were constructed to illustrate differences in soil bacterial and fungal communities between less continuous planted (LCM) plot systems (1Y and 3Y) and multiple continuous monoculture (MCM) plot systems (5Y and 10Y) in three compartments (Figures 3, 4). The network properties of soil bacterial and fungal communities are summarized in Supplementary Table 4. In the bacterial networks, LCM soils and MCM soils resulted in networks with similar sizes in RS (19 and 19 nodes, 31 and 36 edges, respectively; Figure 3 and Supplementary Table 4). MCM soil [1 (RP) and 3.157 (RT)] had a lower value of average path length (path length is the shortest path between two nodes) than the LCM soils [2.502 (RP) and 4.658 (RT)]. The higher modularity value indicates the denser connections between the nodes within the module. The results showed that in both RS and RP rhizocompartment, the modularity value of MCM soil (0.773 and 0.917, respectively) was higher than the value in LCM soil (0.765 and 0.533, respectively). The RS and RT were mainly composed of *Proteobacteria*, while the RP was mainly *Proteobacteria* and *Firmicute* (Figure 3). The keystone bacterial genera in these systems are listed in Table 1. It is worth mentioning that unidentified *Nitrosomonadaceae*, *Bacillus*, *Fictibacillus*, *Lysinibacillu*, *Bradyrhizobium*, *Shinella*, and *Herbaspirillum* occupied an important position in MCM soil.

**TABLE 1:** The keystone genera in the networks of bacterial and fungal communities.

	RS1Y-3Y	RS5Y-10Y	RP1Y-3Y	RP5Y-10Y	RT1Y-3Y	RT5Y-10Y
Bacteria	*Lysobacter*	*Acidibacter*	*Pseudoduganella*	*Novosphingobium*	*Rhodoplanes*	*Mesorhizobium*
	*Pseudoxanthomonas*	*Steroidobacter*	*Novosphingobium*	*Steroidobacter*	*Permianibacter*	*Bradyrhizobium*
	*Arenimonas*	*Novosphingobium*	*Paenibacillus*	*Bacillus*	*Stenotrophomonas*	*Woodsholea*
	*Nordella*	*unidentified_Nitrosomonadaceae*	*Lysinibacillus*	*Fictibacillus*	*Nannocystis*	*Shinella*
	*Bradyrhizobium*	*Halomonas*	*Fictibacillus*	*Lysinibacillus*	*Massilia*	*Herbaspirillum*
Fungi	*Mortierella*	*Lulwoana*	*Alternaria*	*Sarocladium*	*Microidium*	*Coprinopsis*
	*Cryptococcus*	*Geosmithia*	*Cladosporium*	*Ochroconis*	*Pseudozyma*	*Lentinula*
	*Pyrenochaetopsis*	*Basidiobolus*	*Gibberella*	*Fusicolla*	*Sarocladium*	*Anomoporia*
	*Enterographa*	*Metarhizium*	*Elaphomyces*	*Lectera*	*Talaromyces*	*Thanatephorus*
	*Hannaella*	*Mortierella*	*Lactarius*	*Bullera*	*Exserohilum*	*Tricholoma*

With respect to fungal networks, the nodes and edges of MCM soils were 16 and 12 (RS), 20 and 13 (RP), and 11 and 7 (RT) higher than that 16 and 8 (RS), 8 and 4 (RP), and 8 and 4 (RT) in LCM soil, respectively, indicating that MCM soils had more connections compared to LCM soil. The values of network modularity presented an opposite trend in which the values [0.875 (RS) and 0.75 (RT)] in LCM soil were higher than those in MCM soils [0.708 (RS) and 0.735 (RT)] (Supplementary Table 4). The results from Figure 4 showed that most genera (more than 80%) in three compartments of LCM soil belonged to *Ascomycota* and *Basidiomycota*. While all genera were assigned to four phyla and various phyla accounted for a different proportion in MCM soils. *Mortierella* species had the highest level of abundance compared to other fungi. Further, these networks were not associated with *Fusarium*.

The results showed that there were correlations between soil physical–chemical properties and the keystone species. Among them, *Pseudoxanthomonas*, *Altererythrobacter*, unidentified *Gammaproteobacteria*, *Pseudolabrys*, and *Hirschia* showed a significant positive correlation with AN, TN, and AP (Supplementary Figure 4). *Novosphingobium*, *Gaiella*, *Halomonas*, and *Nordella* showed a significant negative correlation with AN, TN, and AP (Supplementary Figure 4). With respect to fungi, *Geosmithia*, *Talaromyces*, and *Guehomyces* showed a significant negative correlation with TK (Supplementary Figure 5). *Sporobolomyces* and *Sarocladium* showed a significant positive correlation with TK (Supplementary Figure 5).

### Abundance of Specific Microbes by Quantitative PCR

**FIGURE 5:**
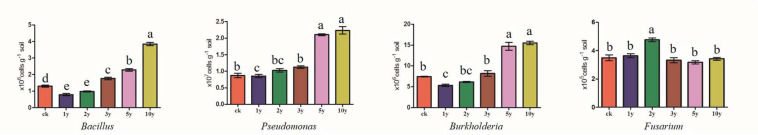
Quantification of total bacteria, total fungi, and specific microbes (including *Bacillus*, *Pseudomonas*, *Burkholderia*, and *Fusarium*) in different soil samples. Small letters in figures show significant differences determined by Tukey’s test (*p* ≤ 0.05, *n* = 3).

The qPCR analysis showed that the abundance of total bacteria, *Bacillus*, *Pseudomonas*, and *Burkholderia* spp., were significantly greater in the 5Y and 10Y (strong allelopathic activity soils) than in the 1Y and 2Y, while total fungi and *F. oxysporum* showed the other trend and tended to stay at a stable level with an increase of continuous cropping years (Figure 5). The results were consistent with high-throughput sequencing analysis (Figure 2).

### Isolation and Functional Verification of Key Microorganisms

Among the cultured organisms, there were significantly higher occurrence of *F. solani* and *F. oxysporum*. Tests demonstrated that the tissue culture of *A. bidentate* under *F. oxysporum* treatment exhibited wilt disease symptom (Supplementary Figures 6a,b). Then, the antagonistic assay of the purified isolated *Bacillus* spp. against *F. solani* and *F. oxysporum* was evaluated. In Supplementary Figure 6c, the bacteria exhibited a strong antagonism against the two fungi. After molecular identification, the strains were aligned with *B. amyloliquefaciens* 4 (BA4), *B. subtilis* 35 (BS35), *B. subtilis* 74 (BS74), and *B. halodurans* 75 (BH75). Subsequently, the bacteria were added and inoculated to the pots where the *A. bidentata* were grown. Both the root length and the fresh weight of *A. bidentata* under treatment by BS 74 and BH 75 were significantly (*p* < 0.05) greater than the control (Supplementary Figure 7).

### Identification and Analysis of Main Phytosterones in Mediums and Its Interaction With Microbes *in vitro*

The results showed that phytosterone levels tended to increase as the growth of the tissue culture plant increased. In particular, the content of β*-ecdysterone* after 373 and 396 days (2.34, 1.84 μg/ml media) tissue culture was 6.3 and 4.9 times higher than after 140 days (0.37 μg/ml), respectively (Supplementary Figure 8a). Although it was found that no phytosterone was detected in 1Y and CK soils, the content of β*-ecdysterone* did not accumulate in consecutively monocultured soil (Supplementary Figure 8b). This may be the result of the combined effect of root exudation intensity and soil microbial catabolism in monocultured soil.

**FIGURE 6:**
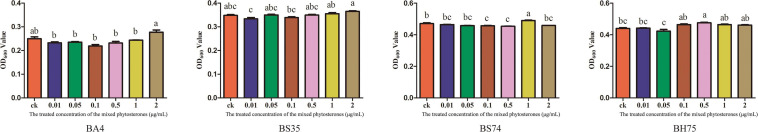
The effects of a mixture of phytosterone on the growth of beneficial bacteria [including *Bacillus amyloliquefaciens* 4 (BA4), *Bacillus subtilis* 35 (BS35), *Bacillus subtilis* 74 (BS74), and *Bacillus halodurans* 75 (BH75)]. Small letters in figures show significant differences determined by Tukey’s test (*p* ≤ 0.05, *n* = 3).

**FIGURE 7:**
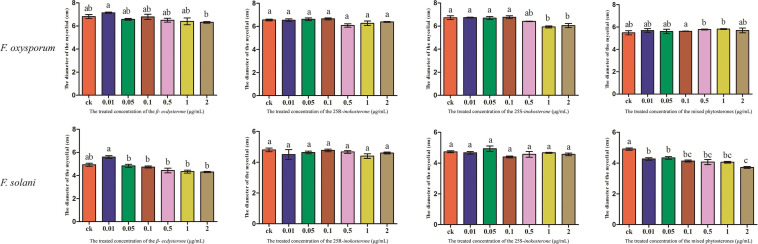
The effects of single (β-*ecdysterone*, 25R-*inokosterone*, and 25S-*inokosterone*) and a mixture of phytosterone on the mycelial growth of *F. oxysporum* and *F. solani*. Small letters in figures show significant differences determined by Tukey’s test (*p* ≤ 0.05, *n* = 3).

Further, the effects of phytosterone allelochemicals in root exudates on the growth of beneficial bacteria (BA4, BS35, BS74, and BH75) and pathogenic fungi (*F. solani* and *F.* oxysporum) were determined (Supplementary Figures 6, 7, 9). A mixture of phytosterones significantly promoted the growth of beneficial bacteria especially BA74 and BH75 (Figure 6). Among the three phytosterone compounds identified in the soil, β*-ecdysterone* displayed the most stimulatory effect. What is more, mycelial growth of *F. solani* was inhibited by a mixture of phytosterones (Figure 7). Likewise, the various single phytosterones displayed different influences on the mycelial growth.

## Discussion

It can be clearly seen that both plant and soil environment provided an ideal habitat for some microorganisms, and these species had a great impact on plant productivity (Van der Heijden et al., 2008; Schlaeppi and Bulgarelli, 2015). Among them, the role of the microbiome including bacteria and fungi must be taken into consideration. On the one hand, soil microbes are important positive catalysts of plant productivity (Raaijmakers et al., 2002). Precisely, mechanisms through which microorganisms can enhance plant productivity have been identified and include triggering inducing systemic resistance by priming for accelerated defense-related gene expression (Van Der Ent et al., 2009), fixation of nitrogen from atmospheric N (Unkovich et al., 2008), and solubilization of nutrients that are inaccessible to plant roots (Zahir et al., 2003). On the other hand, soil pathogens grow rapidly to obtain sufficient numbers on their host and be able to infect host tissue resulting in a reduction of plant biomass (Raaijmakers et al., 1995; Beattie, 2006).

The data presented here provide a characterization of the bacterial and fungal community in three root-associated layers of *A. bidentata*. The structure, composition, and microbial abundance of soil fungal and bacterial communities differed in relation to different consecutive monoculture years. From the viewpoint of diversity indices, it could be shown that the diversity decreases from rhizosphere to rhizoplane and then to root for both bacteria and fungi. The shifts in community structure from rhizosphere toward roots was a result of a three-step selective filter by each of these compartments (Edwards et al., 2015; Beckers et al., 2017). From the viewpoint of structure and composition, the various proportions of certain microbes (such as *Sphingomonas*, *Bacillus*, and *Pseudomonas*) across the three rhizocompartments in our study indicated that microbes were attracted to suitable niche. When rhizoplane microbiome data were taken into consideration, the most dominant genus, *Bacillus* spp., exhibited higher relative abundance in the MCM soils compared to LCM soils. This selection by the plant may be explained by the fact that this bacterium was known to have the ability to form biofilms that helped to generate antagonistic effects on pathogenic fungi (Chen et al., 2013). With respect to the root, *Pseudomonas* spp. became the dominant bacterium with the same trend of *Bacillus* spp. in different continuous cropping samples. The change from rhizoplane to root further clarified that *Bacillus* spp. when bound to the rhizoplane served as a critical gating role. Moreover, other studies found that *Pseudomonas* isolates from the endosphere engaged in more metabolic pathways for plant signaling compounds, bacterial–plant interactions, and root growth promotion when compared to isolates obtained from the rhizosphere (Timm et al., 2015). The roles of specific species of *Pseudomonas* app. need to be discussed in-depth in future research. The results reported here also showed that the relative abundance of *Proteobacteria* was increased in root compared to rhizosphere soil, which was similar to studies of *Arabidopsis* and rice (Bulgarelli et al., 2012; Edwards et al., 2015). This suggested that the distribution of different bacterial phyla inside the roots might be similar for many land plants. The plant cell-wall feature provided a cue that this *A. bidentata*-unspecific root-inhabiting microbiota might play a vital role in the decomposition of organic matter after plant death (Bulgarelli et al., 2012). Therefore, the niche-specific settings among different rhizocompartments can exhibit different eco-niche functions in an ecosystem.

By employing network analysis, new insights were revealed regarding the bacterial and fungal microbiota associated with different consecutive monocultures. Twelve network graphs were constructed to display interactions among different genera. The bacterial community was found to have a greater number of functionally interrelated members (231 nodes and 402 edges) than those in the fungal community (79 nodes and 48 edges). The ecosystem characteristics and processes were determined by interactions among species that are able to modify energy pathways or the abundance of species, not simply the presence of species (Chapin et al., 2000; Faust and Raes, 2012). Our results suggest that bacteria may play a more important role in continuous cropping systems. For the bacterial network, the keystone bacterial species were unidentified: *Nitrosomonadaceae*, *Bacillus*, *Fictibacillus*, *Bradyrhizobium*, *Shinella*, and *Herbaspirillum*. In addition, most of these functional bacteria exhibited different eco-niche functions in the soil ecosystem, which have been shown to be involved in plant growth-promoting activities including production of different plant hormones, soil nutrient bioavailability, and generating antagonistic effects (Han et al., 2014; Li B. et al., 2014). It is worth mentioning that there were many nitrogen-fixing bacteria (including *Bradyrhizobium*, *Shinella*, and *Herbaspirillum*), which are responsible for the nitrogen input into various ecological niches in this system (Perret et al., 2000; Elbeltagy et al., 2001; Lin et al., 2008). For example, Baldani et al. (1986) successfully isolated and verified nitrogen fixation activity of *Herbaspirillum* from cereal roots. For fungi, *Mortierella* were the most prevalent genus in this system. Previous studies have shown that some species of *Mortierella* are antagonistic to pathogens, and several isolates have been determined to be potential antagonistic agents against major potato scab pathogens (Tagawa et al., 2010). However, *Fusarium* was absent in the network graph, which was the opposite observation to that obtained from previous reports on the monoculture of medicinal plants (*R. glutinosa* and *R. pseudostellariae*) and disease-conducive soil (Wu et al., 2015, 2016; Xiong et al., 2017). Previous studies have shown the significant correlations between key environmental factors (such as available nutrients, soil pH, soil texture, and climatic conditions) and the keystone soil microbes (Fierer and Jackson, 2006; Banerjee et al., 2016). The vital participation of soil microbiomes in soil nutrient cycling was also reported previously, especially in nitrogen fixing and phosphate solubilization, which were key steps for promoting plant growth (Lugtenberg and Kamilova, 2009; Banerjee et al., 2018). In this study, our result provided evidence that AN, TP, and AP contents were closely related to bacterial community while TK was correlated with fungal community. The suitable eco-niche differentiation in each compartment and beneficial plant microbiome interactions are avenues to help plant growth and development (Compant et al., 2019). It was therefore suggested that the recruitment of beneficial microorganisms and disappearance of harmful microorganisms may in turn contribute to a higher disease-suppressive ability of the specific medicinal plant *A. bidentata* to adapt to the consecutive monoculture system. This would be different from most medicinal plants severely suffering from replanting disease in the monoculture system.

In addition, plants along with their root system can provide a unique ecological niche for soil microbiota and transport up to 40% of their photosynthates to the soil in order to feed microorganisms, which are mostly carbon starved in the soil (Bais et al., 2006; Garbeva et al., 2011). The evidence that intercropping systems or rhizosphere soil have a higher microbial population density indicates that plant species are able to affect species-specific shift as well as the composition of microbial community through the production of specific root exudates (Berg et al., 2006; Li L. et al., 2014). In the present study, we found that the phytosterone produced by tissue culture plantlet roots of *A. bidentata* accumulated in the culture medium. However, phytosterone did not accumulate in the rhizosphere soil over all the years of monoculture. Therefore, we propose that root exudates were involved in biological processes involving microbial metabolism. The experimental results also showed that exogenously added phytosterones could significantly promote the growth of beneficial bacteria especially *Bacillus* 4 and *Bacillus* 75 and inhibit mycelial growth of *F. solani* and *F. oxysporum*. An increasing number studies have shown that root exudates could select microorganisms in the rhizosphere, which manifested as an increased density of pathogenic *F. oxysporum* and a low abundance of beneficial bacteria (Zhou and Wu, 2012; Li X. et al., 2014). It should be noted that the positive or negative effects of root-released allelochemicals in our study displayed an opposite trend from previous studies. Furthermore, certain doses of phytoecdysteroid were able to cause abnormal molting, immobility, weakened invasion, impaired development, and death of nematodes on plants, which resulted in protecting spinach from plant-parasitic nematodes (Soriano et al., 2004). Cui et al. (2015) found that a 1% dose of plant *ecdysterone* could increase the number of probiotic *C. coccoides* in the intestine, and a 2% dose increased the proportion of *Enterobacteriaceae*. The ideal population structure with a higher abundance of beneficial microorganisms (*Bacillus*) but lower abundance of harmful microorganism was influenced by the phytoecdysteroid exudates, which may partially account for the healthy growth of *A. bidentata*.

**FIGURE 8:**
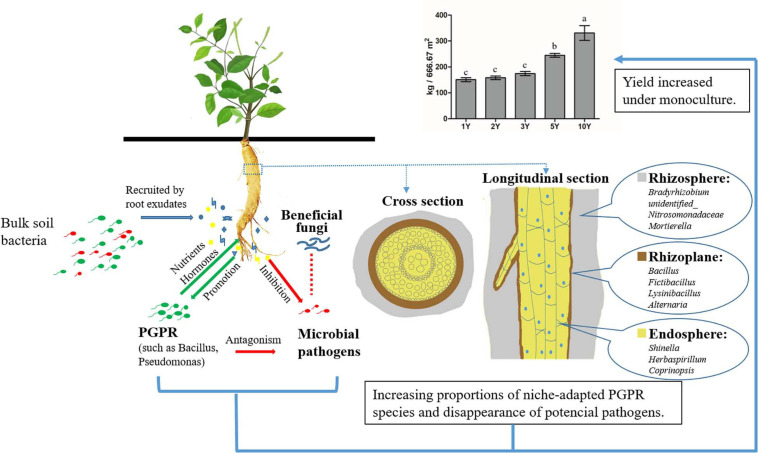
Schematic representation of microbiome interactions and eco-niche distribution in three rhizocompartments of *A. bidentata* in a consecutive monoculture system. The red dotted line represented that no experiments have been obtained *in vitro*.

In summary (Figure 8), we provide an insight into soil bacterial and fungal community structure in three root-associated layers of *A. bidentata* and found key microorganisms as being affected by long-term monoculture *via* network interactions, which included both basic inventory descriptions of species richness and abundance and coexistence and modularity among populations in the microbial community. Our results showed that the MCM soil apparently generated some distinct bacterial taxa such as plant growth-promoting bacteria and nitrogen-fixing bacteria inhabiting the corresponding root-associated layers. However, there were no potential pathogens in the system. Root exudate phytosterones were found to play important roles in the formation of this structure resulting in the presence of more plant-beneficial bacteria and fewer pathogenic fungi, indicating that different plants can shape the microbial community *via* secreting a variety of substances. These findings provide a clue necessary to open a new avenue for modulating the root microbiome to enhance medicinal plant production and sustainability. The key microorganisms obtained in this study can lay the foundation for the development of microbial fertilizer in the future. At the same time, the results also have important theoretical significance and potential application value to elucidate the underground interaction mechanism of *A. bidentata* resistant to continuous cropping for solving the “sick soil syndrome” of most medicinal plants. However, we still need to be aware that further experiments are required to elucidate the intrinsic mechanism of interaction between specific microbes and plant root exudates leading to the tolerance to consecutive monoculture of *A. bidentata*.

## Data Availability Statement

The datasets presented in this study can be found in online repositories. The names of the repository/repositories and accession number(s) can be found below: https://www.ncbi.nlm.nih.gov/, PRJNA606926.

## Author Contributions

SL and WL conceived and directed the project. JW, YL, TC, and XQ did all of the experiments. JW, LW, and HW performed the integrated data analysis. JW and WL wrote the manuscript. CR and PL provided linguistic and scientific assistance. All authors contributed to the article and approved the submitted version.

## Conflict of Interest

The authors declare that the research was conducted in the absence of any commercial or financial relationships that could be construed as a potential conflict of interest.

## Footnotes

1 http://qiime.org/scripts/split_libraries_fastq.html

2 http://drive5.com/uparse/

3 http://www.arb-silva.de/
