# Supplementary material for: Revealing Microbiome Structure and Assembly Process in Three Rhizocompartments of Achyranthes bidentata Under Continuous Monoculture Regimes
Source: Front Microbiol. 2021 Jun 14;12:677654. doi: 10.3389/fmicb.2021.677654 (PMC8236951; doi:10.3389/fmicb.2021.677654)
Supplement: Supplementary file 1 [file Data_Sheet_1.docx]

**Revealing Microbiome Structure and Assembly Process in Three Rhizocompartments of *Achyranthes bidentata* under Continuous Monoculture Regimes**

Juanying Wang^1,2^, Hongmiao Wu^1,2^, Linkun Wu^1,2^, Ye Liu^1,2^, Puleng Letuma^3^, Xianjin Qin^2,4^, Ting Chen^1,2^, Christopher Rensing ^5^, Sheng Lin^1,2*^, Wenxiong Lin^1,2*^

^1^Fujian Provincial Key Laboratory of Agroecological Processing and Safety Monitoring, College of Life Sciences, Fujian Agriculture and Forestry University Fuzhou 350002, Fujian, China

^2^Key Laboratory for Genetics, Breeding and Multiple Utilization of Crops, Ministry of Education / College of Crop Sciences, Fujian Agriculture and Forestry University, Fuzhou 350002, Fujian, China

^3^Crop Science Department, National University of Lesotho, Maseru 100, Lesotho.

^4^College of Crop Sciences, Fujian Agriculture and Forestry University, Fuzhou 350002, Fujian, China.

^5^Fujian Provincial Key Laboratory of Soil Environmental Health and Regulation, College of Resources and Environment, Fujian Agriculture and Forestry University, Fuzhou 350002, Fujian, China.

^*^**Corresponding author:** Sheng Lin or Wenxiong Lin

School of Life Sciences,

Fujian Agriculture and Forestry University,

Fuzhou 350002, China

Phone: (86)-0591-83737535

Fax: (86)-591-83769440

Email: linsh@fafu.edu.cn, lwx@fafu.edu.cn

Table S1. The contents of major elements in rhizospheric soils of *A. bidentata* monocultured for different years

|  | **Total N**  **(g/kg)** | **Total P**  **(g/kg)** | **Total K**  **(g/kg)** | **Available N**  **(mg/kg)** | **Available P**  **(mg/kg)** | **Available K**  **(mg/kg)** | **pH** |
| --- | --- | --- | --- | --- | --- | --- | --- |
| **CK** | 0.5743a | 0.3615b | 6.9493a | 36.6333c | 55.1816ab | 105.3040a | 8.2200d |
| **1Y** | 0.6310a | 0.2466c | 6.9164a | 36.4000c | 49.5512b | 95.6604c | 8.4500c |
| **2Y** | 0.6163a | 0.2211c | 6.6439b | 33.9500c | 49.2452b | 83.0235d | 8.7300a |
| **3Y** | 0.6001a | 0.2383c | 6.6166b | 35.0000c | 52.4684b | 82.1733d | 8.6100ab |
| **5Y** | 0.7219a | 0.4324a | 6.6173b | 77.5833a | 62.8111a | 100.9364b | 8.5600bc |
| **10Y** | 0.7075a | 0.3214b | 6.2742c | 56.7000b | 54.6716b | 85.6441d | 8.6267ab |

Different letters in columns show significant differences determined by Tukey’s test (*p*≤0.05, n = 3).

Table S2. Calculations of observed species, richness and diversity of bacteria under the different treatments.

| **Root-associated Layers** | **Treatments** | **Number of OTUs** | **Shannon** | **Simpson** | **Chao1** | **ACE** |
| --- | --- | --- | --- | --- | --- | --- |
| **Rhizosphere** | 1Y | 3737.00±197.01a | 10.06±0.13a | 0.99±0.00a | 5243.85±603.62a | 5370.15±439.59a |
|  | 3Y | 3809.00±37.64a | 10.22±0.05a | 0.99±0.00a | 5225.15±466.27a | 5317.01±302.99a |
|  | 5Y | 3952.00±401.43a | 10.26±0.25a | 0.99±0.00a | 5475.50±1046.66a | 5555.57±1005.45a |
|  | 10Y | 3821.67±253.33a | 10.12±0.18a | 0.99±0.00a | 5169.36±1037.21a | 5309.32±985.92a |
|  |  |  |  |  |  |  |
| **Rhizoplane** | 1Y | 4448.67±130.20a | 9.75±0.10a | 0.99±0.00a | 4370.48±128.04a | 4447.71±129.70a |
|  | 3Y | 4207.67±705.71a | 9.87±0.37a | 0.99±0.00a | 4106.52±644.47a | 4185.91±688.40a |
|  | 5Y | 4815.67±27.43a | 10.05±0.08a | 0.99±0.00a | 4659.27±35.02a | 4850.88±38.15a |
|  | 10Y | 3987.33±431.45a | 8.18±0.78b | 0.96±0.02b | 3918.81±439.18a | 4064.29±419.39a |
|  |  |  |  |  |  |  |
| **Root** | 1Y | 362.67±17.62c | 1.21±0.24d | 0.30±0.07d | 362.86±29.53b | 383.03±42.19c |
|  | 3Y | 685.67±30.89ab | 6.35±0.05a | 0.97±0.00a | 679.48±37.40a | 680.40±32.26ab |
|  | 5Y | 719.67±35.91a | 4.53±0.04b | 0.84±0.00b | 742.65±63.62a | 761.59±51.02a |
|  | 10Y | 629.33±38.99b | 3.61±0.06c | 0.64±0.01c | 611.25±55.65a | 627.10±51.64b |

Different letters in columns show significant differences determined by Tukey’s test (p≤0.05, n = 3).

Table S3. Calculations of observed species, richness and diversity of fungi under the different treatments.

| **Root-associated Layers** | **Treatments** | **Number of OTUs** | **Shannon** | **Simpson** | **Chao1** | **ACE** |
| --- | --- | --- | --- | --- | --- | --- |
| **Rhizosphere** | 1Y | 704.67±58.62a | 6.31±0.45a | 0.96±0.02a | 571.52±65.19a | 575.65±68.18a |
|  | 3Y | 759.33±94.00a | 6.01±0.43a | 0.94±0.03a | 637.91±100.44a | 642.88±104.58a |
|  | 5Y | 732.33±31.18a | 5.97±0.40a | 0.94±0.04a | 594.75±39.36a | 602.19±45.56a |
|  | 10Y | 783.00±106.89a | 6.28±1.10a | 0.93±0.09a | 701.11±115.32a | 695.32±110.56a |
|  |  |  |  |  |  |  |
| **Rhizoplane** | 1Y | 561.33±123.14ab | 3.59±0.71a | 0.77±0.06a | 610.60±144.53a | 587.50±161.93a |
|  | 3Y | 654.33±29.77a | 3.93±0.18a | 0.81±0.02a | 644.01±59.36a | 671.50±2.12a |
|  | 5Y | 481.33±91.74ab | 3.32±0.66a | 0.75±0.02a | 558.83±112.55a | 428.50±9.19a |
|  | 10Y | 358.33±102.65b | 3.51±0.96a | 0.78±0.17a | 458.90±146.85a | 361.50±144.96a |
|  |  |  |  |  |  |  |
| **Root** | 1Y | 482.33±76.17a | 4.96±0.15a | 0.91±0.01a | 482.56±75.45a | 471.67±86.04a |
|  | 3Y | 280.33±21.22bc | 2.11±0.09b | 0.45±0.02b | 262.38±12.82bc | 259.27±12.81b |
|  | 5Y | 309.33±83.44ab | 4.47±0.58a | 0.88±0.05a | 287.60±63.08bc | 287.87±58.00b |
|  | 10Y | 112.33±41.40c | 3.77±0.54a | 0.85±0.06a | 112.30±40.48c | 117.42±44.21b |

Different letters in columns show significant differences determined by Tukey’s test (p≤0.05, n = 3).

Table S4. The network properties of bacterial and fungal communities.

|  |  | Network diameter (ND) | Graph density | Average path length (APL) | Avg Clustering coefficient (ACC) | Modularity (MD) | Nodes | Edges |
| --- | --- | --- | --- | --- | --- | --- | --- | --- |
| Bacteria | RS1Y-3Y | 1 | 0.111 | 1 | 1 | 0.765 | 19 | 19 |
|  | RS5Y-10Y | 1 | 0.077 | 1 | 1 | 0.773 | 31 | 36 |
|  | RP1Y-3Y | 5 | 0.108 | 2.502 | 0.799 | 0.533 | 37 | 72 |
|  | RP5Y-10Y | 1 | 0.072 | 1 | 1 | 0.917 | 33 | 38 |
|  | RT1Y-3Y | 13 | 0.058 | 4.658 | 0.663 | 0.692 | 79 | 180 |
|  | RT5Y-10Y | 7 | 0.115 | 3.157 | 0.32 | 0.508 | 32 | 57 |
| Fungi | RS1Y-3Y | 1 | 0.067 | 1 | 0 | 0.875 | 16 | 8 |
|  | RS5Y-10Y | 1 | 0.1 | 1 | 1 | 0.708 | 16 | 12 |
|  | RP1Y-3Y | 1 | 0.143 | 1 | 0 | 0.75 | 8 | 4 |
|  | RP5Y-10Y | 1 | 0.068 | 1 | 1 | 0.852 | 20 | 13 |
|  | RT1Y-3Y | 1 | 0.143 | 1 | 0 | 0.75 | 8 | 4 |
|  | RT5Y-10Y | 1 | 0.127 | 1 | 1 | 0.735 | 11 | 7 |


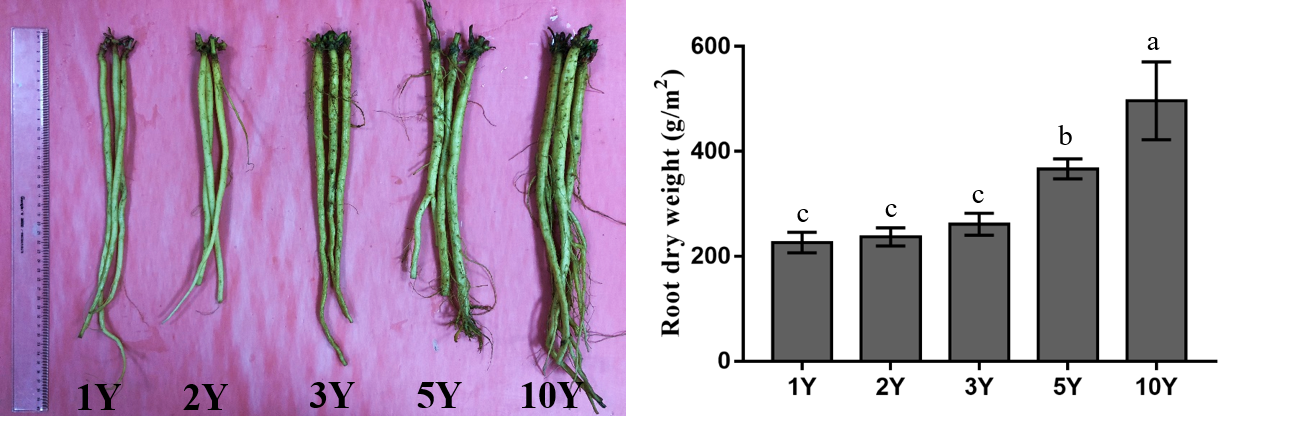


Figure S1. Photograph of below ground and the biomass of *A. bidentata* under different consecutive monoculture years.


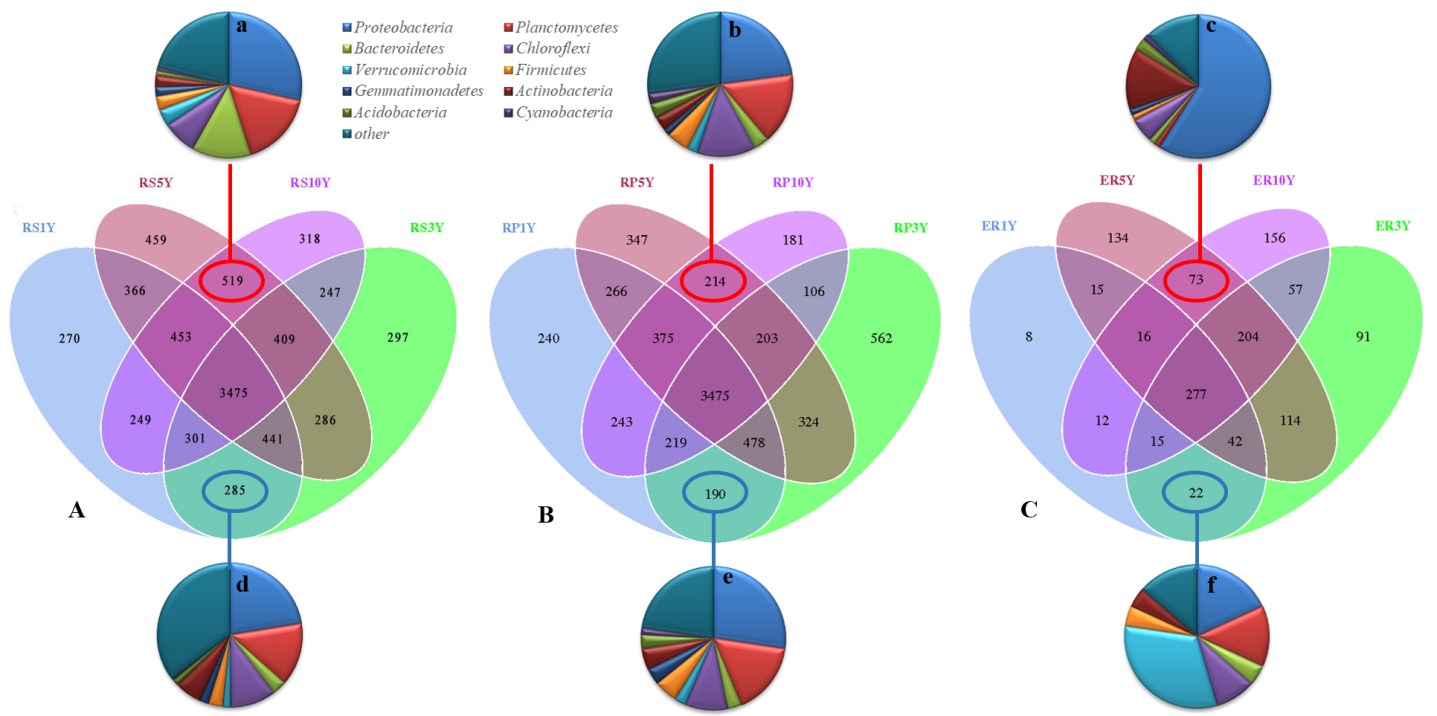


Figure S2. Venn diagrams of shared species-level taxa for bacterial community among the four different types of soil. (A, B and C represented the venn diagram of rhizosphere, rhizoplane and root, respectively. a, b and c Pie charts of exclusive OTUs (519, 214 and 73) shared between 5Y and 10Y based on the average abundance of each phylum. d, e and f Pie charts of exclusive OTUs (285, 190 and 22) shared between 1Y and 3Y based on the average abundance of each phylum.)


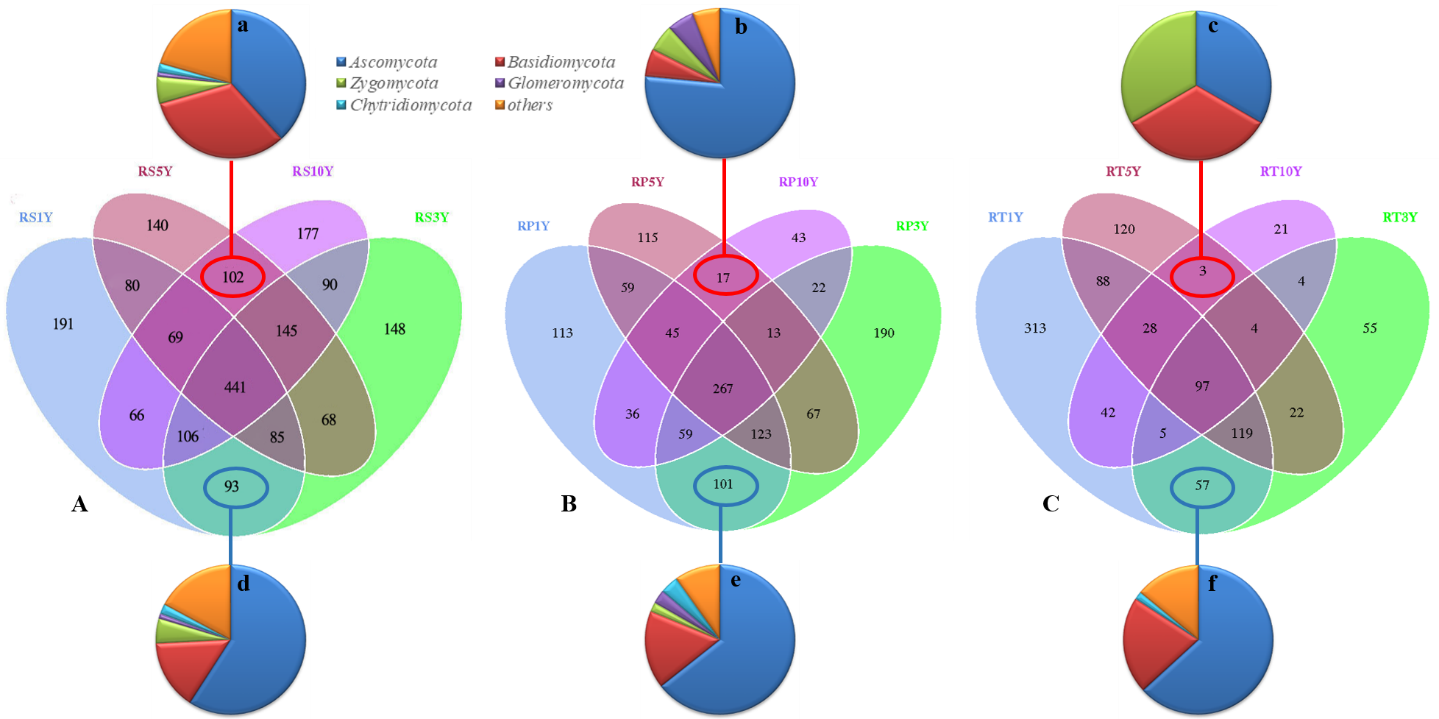


Figure S3. Venn diagrams of shared species-level taxa for fungal community among the four different type soil. (A, B and C represented the venn diagram of rhizosphere, rhizoplane and root, respectively. a, b and c Pie charts of exclusive OTUs (102, 17 and 3) shared between 5Y and 10Y based on the average abundance of each phylum. d, e and f Pie charts of exclusive OTUs (93, 101 and 57) shared between 1Y and 3Y based on the average abundance of each phylum)


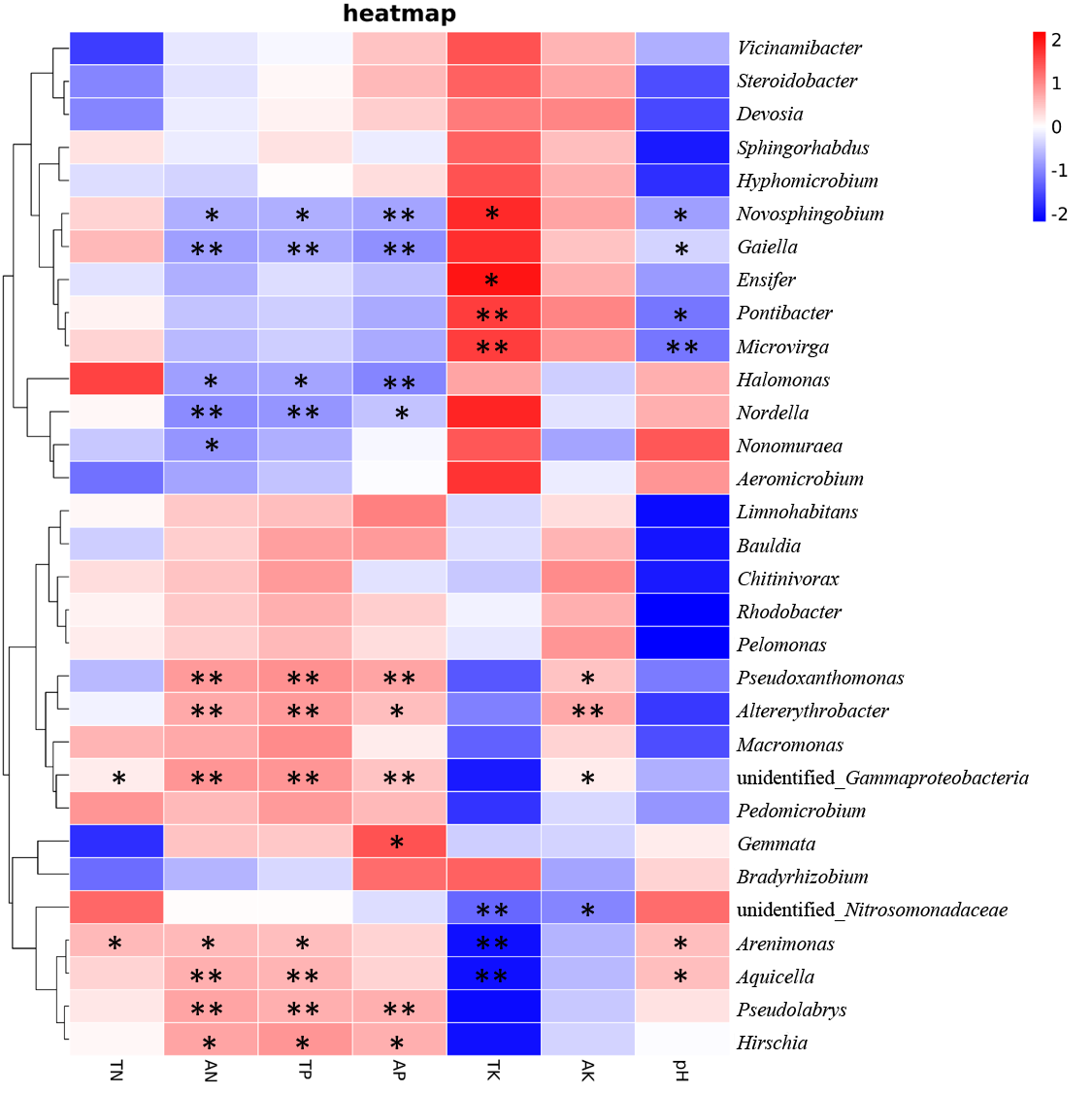


Figure S4. Heatmap of correlation between soil physical-chemical properties and the bacterial keystone genera.


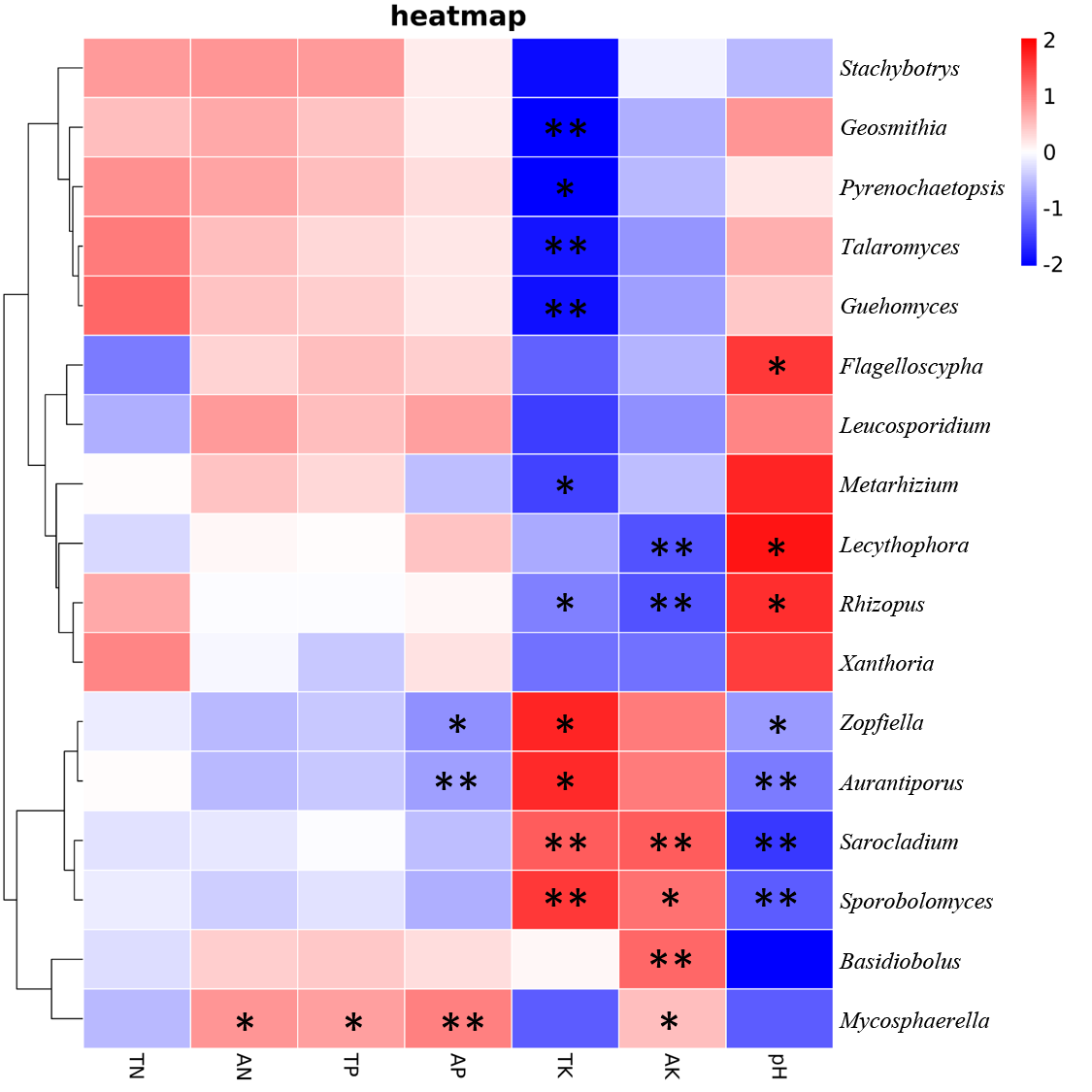


Figure S5. Heatmap of correlation between soil physical-chemical properties and the fungal keystone genera.


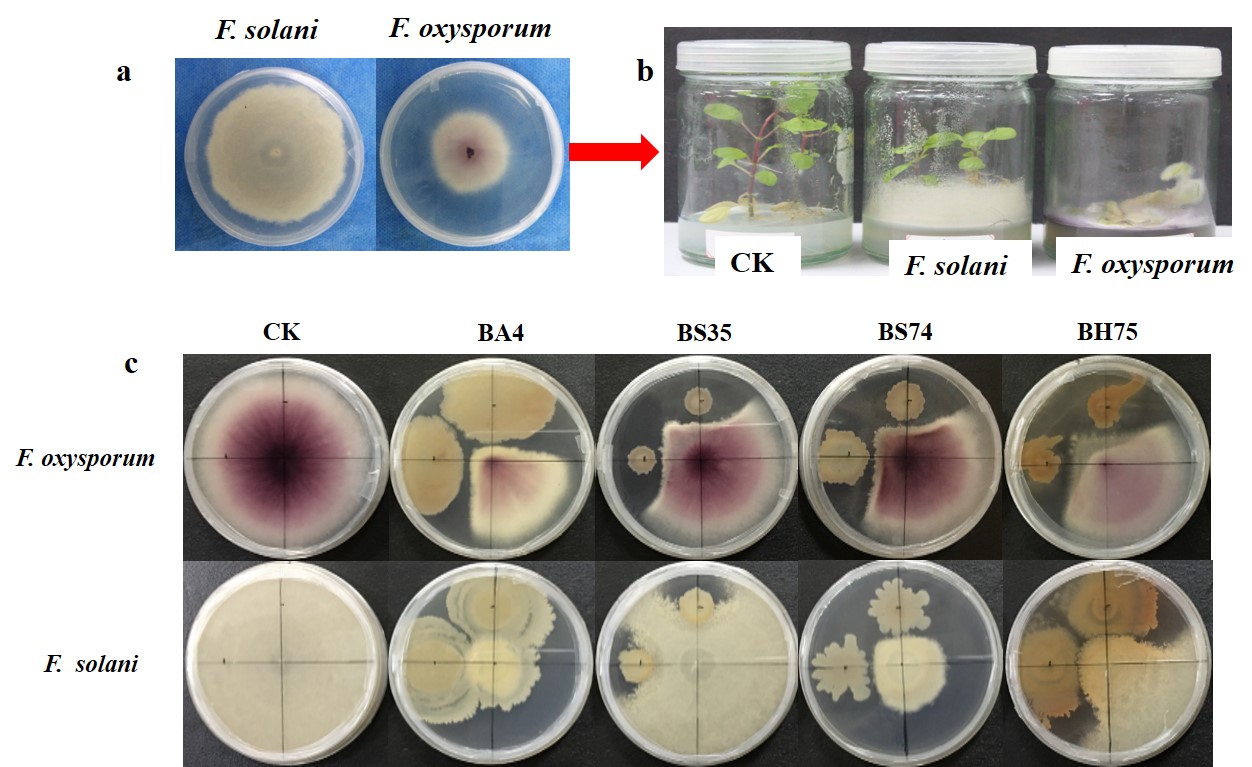


Figure S6. Assessment of the pathogenicity of isolated *F. solani* and *F. oxysporum* (a and b) and the strains with antagonistic activity against *F. solani* and *F. oxysporum* (c).


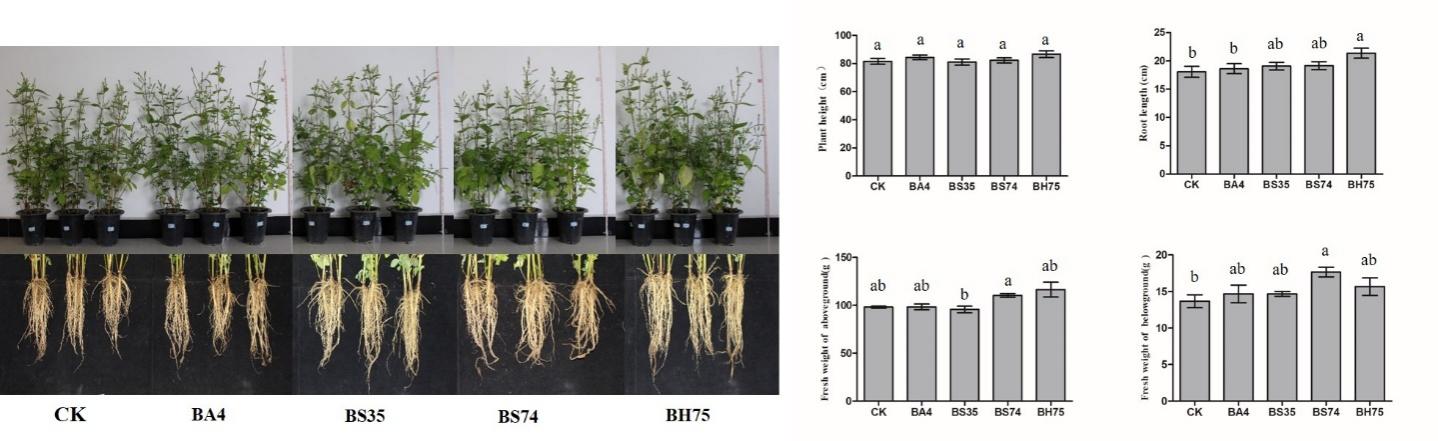


Figure S7. Photographs and biomass of *A. bidentata* under different treatments.


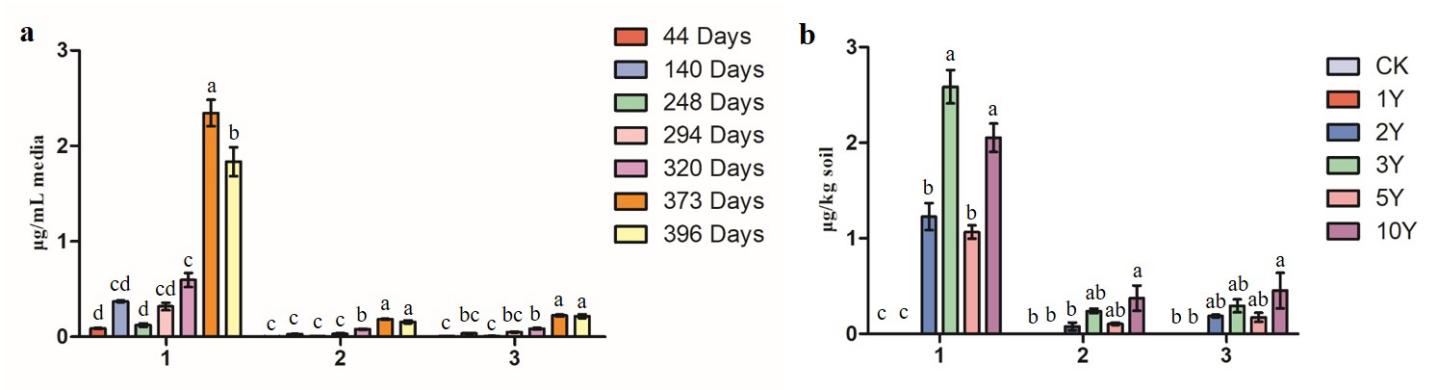


Figure S8. Changes in the content of phytosterones in the tissue culture media (a) and rhizosphere soil (b) of *A. bidentata*. (1, 2 and 3 represented *β- ecdysterone*, 25R-*inokosterone* and 25S-*inokosterone* respectively.)


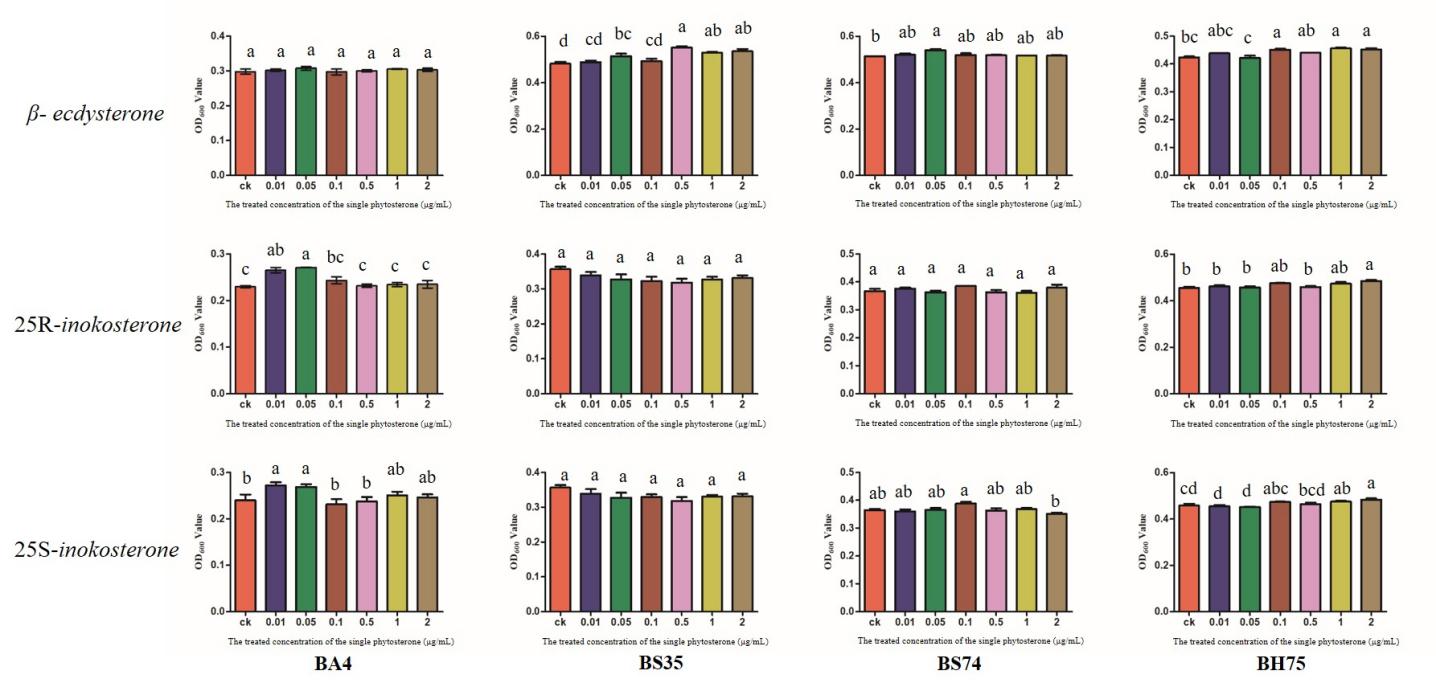


Figure S9. The effects of a single phytosterone on the growth of beneficial bacteria.
